# Midwives’ lived experience of a birth where the woman suffers an obstetric anal sphincter injury - a phenomenological study

**DOI:** 10.1186/1471-2393-14-258

**Published:** 2014-08-03

**Authors:** Malin Edqvist, Helena Lindgren, Ingela Lundgren

**Affiliations:** Institute of Health and Care Sciences, The Sahlgrenska Academy, University of Gothenburg, Arvid Vallgrens backe hus 1, Box 457, 405 30 Gothenburg, Sweden; Department of Women’s and Children’s Health, Karolinska Institutet, Retzius väg 13 A, 171 77 Stockholm, Sweden

**Keywords:** Obstetric anal sphincter injury, Midwives’ lived experiences, Phenomenology, Reflective lifeworld research, Reflection

## Abstract

**Background:**

The occurrence of obstetric anal sphincter injuries (OASIS) has increased in most high-income countries during the past twenty years. The consequences of these injuries can be devastating for women and have an impact on their daily life and quality of health. The aim of this study was to obtain a deeper understanding of midwives’ lived experiences of attending a birth in which the woman gets an obstetric anal sphincter injury.

**Methods:**

A qualitative study using phenomenological lifeworld research design. The data were collected through in-depth interviews with 13 midwives.

**Results:**

The essential meaning of the phenomenon was expressed as a deadlock difficult to resolve between a perceived truth among midwives that a skilled midwife can prevent severe perineal trauma and at the same time a coexisting more complex belief. The more complex belief is that sphincter injuries cannot always be avoided. The midwives tried to cope with their feelings of guilt and wanted to find reasons why the injury occurred. A fear of being exposed and judged by others as severely as they judged themselves hindered the midwives from sharing their experience. Ultimately the midwives accepted that the injury had occurred and moved on without any definite answers.

**Conclusions:**

Being caught between an accepted truth and a more complex belief evoked various emotions among the midwives. Feelings of guilt, shame and the midwife’s own suspicion that she is not being professionally competent were not always easy to share. This study shows the importance of creating a safe working environment in which midwives can reflect on and share their experiences to continue to develop professionally. Further research is needed to implement and evaluate the effect of reflective practices in relation to midwifery care and whether this could benefit women in childbirth.

## Background

The occurrence of obstetric anal sphincter injuries (OASIS) has increased in Sweden during the last twenty years even though there has been a slight reduction in the prevalence from 4.2% to 3.5% in the last few years [1]. The consequences of these injuries can be devastating for women [2] and involve symptoms such as perineal pain, dyspareunia [3] and faecal incontinence [4]. Obstetric anal sphincter injuries also prolong the time from birth until sexual intercourse is resumed [5]. These conditions have impact on women’s daily life and quality of health.

The problem of the increased incidence of OASIS in Sweden has attracted attention from the professions involved as well as the media and there is ongoing discussion within the professions involved regarding the reasons for and ways of preventing severe perineal trauma. Most hospital obstetric departments have created inter-professional training programmes, audits and individual evaluation of midwives and obstetricians to decrease sphincter injuries but no national strategy has been developed.

Most of the research on OASIS has focused on prevalence, risk factors and women’s symptoms after an injury. Furthermore women’s experiences of severe perineal trauma and obstetric anal sphincter injuries have been described. The experience of obstetric anal sphincter injuries during childbirth appears to be a complex phenomenon that affects the women both physiologically and psychologically and alters the women’s understanding of their identity as sexual beings [2].

In Sweden midwives are the primary caregivers during pregnancy, birth and in the postnatal period. They handle uncomplicated births independently, while obstetricians are responsible for complicated deliveries, such as forceps and vacuum extractions and for suturing severe vaginal tears and sphincter injuries. The midwives’ experience when the woman they attend suffers an obstetric anal sphincter injury is less studied. The aim of this study was to obtain a deeper understanding of midwives’ lived experience of a birth when the woman gets an obstetric anal sphincter injury.

## Method

To obtain a deeper understanding of the midwives’ experiences of attending a birth in which the woman suffered an obstetric anal sphincter injury, a phenomenological lifeworld research design was used. The reflective lifeworld design developed by Dahlberg [6] is based on the work of philosophers Husserl, Heidegger, Merleau-Ponty and Gadamer. Phenomenology, as in reflective lifeworld research, seeks patterns of meanings of experience, the structures as well as unique experience through going to the things themselves [6]. The life-world is moreover the lived world that human beings access through the subjective body. The world is understood through our bodies as we use our bodies to mirror the world [7]. Reflective lifeworld research is well designed to investigate phenomena as they are presented in daily life, which in this context are midwives’ experiences of attending a birth in which the woman suffered a sphincter injury.

A central idea in reflective lifeworld research design is to assume a phenomenological attitude, which requires openness toward the phenomenon chosen for study throughout the process. The researcher’s own knowledge and experience of the phenomenon has to be bridled. Bridling can be understood as constantly questioning and reflecting upon the researcher’s preunderstanding of the phenomenon [6].

### Settings and participants

For the purpose of this study, 13 midwives were interviewed. The interviews were conducted with midwives in three different delivery wards, one in Stockholm and two in Gothenburg. In Stockholm the participating midwives cared for women with both low-risk labors and an increased risk who needed specialized obstetric care. The midwives interviewed in Gothenburg worked at units where midwives only care for women with low-risk pregnancies and expected normal labour.

Inclusion criteria for this study were that the midwife had recently assisted a woman who gave birth spontaneously and suffered a sphincter injury. A pilot study showed that to get the midwives’ lived experience of the matter it was of importance to conduct the interview before the memory of the incident faded. When the birth had taken place relatively recently the descriptions in the interviews were richer and included more nuances, which is essential in this research design (Dahlberg, 2006). The interviews with the midwives were conducted within a timespan after the birth (where the woman sustained her trauma) from approximately two weeks to six months. Assisted vaginal births by vacuum extraction or forceps were excluded since in most cases such births are conducted by obstetricians.

The midwives interviewed in Stockholm were found in the delivery ward’s database and in Gothenburg by a search through the delivery ward’s logbook. A variation in working experience was sought. The midwives’ clinical experience of working in a delivery ward varied from 3 months to 15 years (median 7 years) though some had been midwives longer but in other areas of midwifery practice. Two midwives in the delivery ward in Stockholm who knew about the study approached the researcher to be included, as they had recently experienced a birth where the woman suffered a sphincter injury. Six midwives chose to be interviewed at home and seven chose to be interviewed in a secluded room in the delivery ward. One midwife declined to participate due to personal circumstances.

### Ethical considerations

The study was approved by the local Swedish Research ethics committee (nr 385-12). The midwives were contacted by phone and had the option to decline participation. They were also given time to consider whether they wanted to participate. If they seemed hesitant the researcher and the midwife made an agreement as to whether to call later and if so after how long. The midwives gave their written and informed consent before the interviews. They were informed that the data would be handled with confidentiality, that participation was voluntary and that they had the right to withdraw their consent at any time. The participants were offered the opportunity to read the transcripts of their interview, though none of them chose to. Names used in reporting the data are pseudonyms.

### Collection of data

The interviews started with an open question: Can you tell me about this birth where the woman experienced an anal sphincter injury? Clarifying questions were asked when needed and an immediacy in the interview context was sought [6]. These questions were asked to obtain a deeper understanding of each midwife’s experience, for an example: Can you tell me more… Could you explain this further… The interviews took this relatively recent experience as their starting point and an in-depth interview which involved the midwives’ lived experience of the matter could then ensue.

### Data analysis

The data were analyzed in line with reflective lifeworld research as described by Dahlberg et al. [6]. The interviews were recorded and transcribed verbatim and they were read and re-read several times to reach an initial first understanding of the text and to get a sense of the whole. The next step in the analysis included extracting meaning units [8] from the text according to the aim of the study. The meaning units were organised into different clusters and through the different clusters and the text as a whole the essential structure of the studied phenomenon emerged. This process meant going between the parts of the text and the text as whole to see the essence emerge as a new whole. The essential structure was further described by its constituents (i.e. the meaning that constitute the actual essence), to show all the nuances and variations of the studied phenomenon. Throughout the analysis there has been a constant and continuous control to detect any inconsistency between the parts of the analyzed text and the whole. The process of abstracting the essence and finding the constituents has been conducted as an ongoing discussion between the authors in the research group.

## Results

### Essential structure

The essential structure of being a midwife when the woman suffers an obstetric anal sphincter injury during childbirth is to confront a truth, which is perceived as well known among midwives. This truth is that a skilled midwife can prevent severe perineal trauma. At the same time the midwife has to relate to a more complex belief, which implies that sphincter injuries cannot always be avoided no matter what the midwife does during the active phase of the second stage and the birth of the baby.

When the midwives try to relate to both this accepted truth and the more complex belief at the same time they can find themselves in a deadlock, which is difficult to resolve. This deadlock involves time and space, from the active phase of the second stage where the presenting part is visualised, when the sphincter injury is diagnosed until afterwards when the midwife reflects upon the whole situation and context. The midwife considers whether it would have been possible to act differently in the particular situation but at the same time senses that that there were factors at the time that made it impossible to manage the birth differently.

The accepted truth among midwives that a skilled midwife can avoid severe perineal trauma creates feelings of guilt. When relating only to this accepted truth, the midwives experiences guilt for letting the woman down, questioning their skills as midwives and the professional foothold is lost for a while. When the midwives relate to the more complex belief, they can feel relieved since they did their utmost to avoid the trauma and sphincter injuries are not always avoidable. To be in such a locked position means to doubt which perspective to choose, the accepted truth or the more complex belief. The midwife tries to handle the feelings of guilt by finding a valid explanation for the sphincter injury. If a valid explanation is found there is a way out of the deadlock and the feeling of guilt is diminished.

The experiences of guilt and blaming oneself for what happened may be related to a fear of appearing vulnerable. When judging themselves the midwives are also afraid of being judged by others. This is also part of the deadlock as it stops the midwife from opening up and addressing the feelings connected to the experience and the course events took. To be able to move on the midwife reflects back to find explanations for what happened and to develop professionally, but may feel that there are no answers to the questions. Ultimately the midwife accepts what happened and moves on without any definite answers to the questions why this happened and how it can be prevented from happening again.

The essence can be further described by its six constituents: “to see and evaluate the signs and feel the tear occurring”, “to do everything possible to prevent the injury”, “not seeing any signs and being surprised”, “to seek explanations for why the injury happened”, “coping with guilt and self-blame” and “to accept, move on and forgive oneself”.

### To see and evaluate the signs and feel the tear occurring

The midwives described how during the active phase of the second stage they could see that there were signs of an imminent tear and possible sphincter injury. The midwives emphasized a deviant appearance of the perineum; that it was whitening, appeared rigid, swollen or if there was bleeding from the vagina. At the same time they pointed out that these signs were not easily interpreted or equivalent with a sphincter injury or even a tear.

*And I think it's difficult… I don’t think a whitening perineum always means any greater risk than normal. In other words, I think it’s hard to say – some are extremely tight, but I sometimes find that even perineums that are, you know, extremely tight…it seems, like, really not good, but then it just relaxes and they suddenly give birth, without any…and the perineum’s intact, with no tearing at all. So I think it’s sometimes hard to determine when the woman’s pushing whether she has a perineum that is going to relax or not.*

The midwives judged the situation from different perspectives including signs of an imminent tear but also their prior experience and their philosophy of care. Johanna describes a VBAC (Vaginal Birth after Caesarean) where the woman was being induced. The woman expressed a strong desire for a natural birth without pain relief and augmentation even as she was being induced. There was progress during the night and no augmentation was needed. The woman experienced the urge to bear down as very painful and she could not resist it. Johanna tries to support the woman in every possible way but the only thing that the woman finds bearable was when she sat on the birth seat or in the bath. The woman gave birth in the bath just before the night shift was about to end. Johanna described how the woman’s perineum was very swollen and that it was difficult for her to get a good visualization. She dwells on the fact that she let the woman give birth in the bath, saying this is not the way she usually handles a birth where there are signs of a possible severe perineal injury.

*Yes…yes, I thought for sure it’s going to work out anyway. That’s what I was thinking…because it had been a long time since I last experienced a sphincter injury. But I thought things would be fine, even though it felt like for sure she was going to get a large tear. But then I also thought that it would be a terrible thing…if a woman who give birth without anaesthetics and does it all herself…surely she’ll stay intact, I thought.*

### To do everything possible to prevent the injury – for the woman’s sake but also for your own

The midwives stated that they were afraid that the woman would sustain an anal sphincter injury. They worried about the woman, about her being separated from the baby and her partner when going to the operating theatre but also about the long-term consequences of an injury such as anal incontinence. The midwives’ fear was not only related to the consequences for the women but it was also related to themselves as midwives. Both these fears made them do everything in their power to prevent the tear from happening. The midwives felt that it was a professional failure if the woman had a sphincter injury and some of them also worried about being judged by their colleagues. In one of the delivery units midwives were also examined in retrospect, data were extracted from the register and the midwives were evaluated annually regarding birthing positions and sphincter injuries. The midwives mentioned the department’s statistics and not wanting to be the midwife responsible for adding to a high incidence of sphincter injuries.

*Yes, I feel awful when there’s a sphincter injury, obviously, because…because…I mean simply in the eyes of my peers, and especially in my stats. You don’t want to be part of those statistics at all.*

In their intent to do everything possible to prevent a sphincter injury the midwives described how they tried to perform a hands on perineal technique better or even harder, putting more counter-pressure on both the occiput and the woman’s perineum. They also expressed that they sometimes did things that they did not actually believe would be preventive such as changing their perineal protection or performing an episiotomy. Catherine describes her perineal protection technique:

*I hardly touch the perineum… I rather don’t want to touch the perineum because I believe that it must be really painful for women… So as little hands-on as possible… But when the contraction comes I hold my hand on the baby’s head… so that I feel… so I can slow down the speed of the head if necessary…*

But if she feels that if there are any signs of an imminent tear she will perform the hands-on technique that is the norm in the delivery ward.

*Yes, I think it’s because, because I want to minimise the risk. Yeah…at least, I don’t want to stand there afterwards thinking…yes, I should’ve had my hand there, things might have worked out differently then. It’s more a like feeling of security for myself. That I at least tried to make it so nothing like this would happen.*

The midwives in this study sometimes described how at times they requested assistance from their colleagues if they thought there was a risk that the woman would get a severe perineal injury. A colleague can assist her and perhaps see other solutions or options in the actual situation but the midwife could also ask for assistance for her own sake. If the woman experiences a sphincter injury the two midwives can reflect upon the situation afterwards but the colleague can also serve as a witness, to ensure that everything was done right and according to what is perceived as good midwifery practice.

Hannah’s story is about a woman in labour with her first child. Everything is fine and the woman is pushing without instructions in a kneeling position. Suddenly when the baby’s head starts to crown, Hannah can see a small tear in the middle of the perineum, like a buttonhole. She worries that this tear might extend and she asks the woman to turn around to a semi-recumbent position and asks a colleague for help. Together they decide that the best option is to perform an episiotomy to avoid a sphincter injury but in vain. Afterwards Hannah reflects about asking her colleague for assistance:

*What you’re thinking is that this buttonhole tear could lead to a sphincter injury… and you don’t want a sphincter injury. And that I made sure I had another midwife present…and that the two of us decided to perform an episiotomy. So I somehow still felt that I’d done everything I could to reduce the risk of a large tear. So if it occurs…when a large tear did in fact occur…you could still have a little bit of the feeling that ‘I’ve done everything I could to avoid it’. So then it seemed less like my fault… but rather something that could not have been avoided…*

### Not seeing any signs and being surprised

In contrast to seeing signs and knowing or at least suspecting a severe perineal injury before the examination, there were also times when the injury caught the midwife by surprise. For example, when the midwives felt that the baby was crowning and being born slowly and when they felt that they had a good hands-on technique to prevent any injury from happening. Or when they had not seen any signs of an imminent tear or risk factors during the second stage.

*No, because I felt things were going so smoothly, the birth was still going smoothly, and I applied a really firm hands-on technique and guided the baby out, so I thought ‘No…no, that was not what was in my mind at all’, afterwards.*

Signs that worried the midwives or those they paid attention to during the second stage were often related to the appearance of the perineum. When the midwives reflected on the situation afterwards other factors were also analysed, such as how they had managed the active phase of the second stage, the birthing position, wondering if an episiotomy would have made any difference or whether they had forced the second stage too much.

*It was unexpected, it was…It’s always easy when you ‘replay the tape’ and start thinking ‘but why the hell would I put her into the lithotomy position, why the sudden rush?’ I guess that was stupid… so I guess that’s what I feel…that you need to heed the signs and…I guess I should have been able to, like, do things in a different way…*

In cases in which the midwife had not seen any signs of an imminent tear she could reflect afterwards on the woman’s predisposition to sustaining a sphincter injury

*Spontaneously I feel: ‘What are their tissues like?’ You can have a woman has been in labour and pushing for a long time…with a large-size baby… and she doesn’t suffer the slightest scratch… And another time you can see no risk factors and still she ends up with a sphincter injury… every case is different…*

### To seek explanations for why the injury happened

After the injury was diagnosed the midwives went through the experience and scrutinised their actions. The first question they asked themselves was: What could I have done differently? They also searched for a reason for the injury happening and balanced between how they managed the second stage against the signs they could see when reflecting afterwards, which they did not focus on during the birth.

*Yes, as for me, in this situation I was a bit angry at myself for not changing her position. 'Cause that could have made a difference… yes… change from lithotomy position and perhaps let her birth on her side. I’ve done that before. It works much better then…but… Yeah, actually, I could have done that.*

Even if the midwives could see that they could have acted differently during the birth, they still felt that it was not necessarily the case that another way of managing the birth would have prevented the injury.

*You want to know why it happened…what was the actual cause? And you’ll probably be wondering about that forever, you’ll never really know…there may have been several causes, and you’ll never know with certainty…*

In certain situations the midwife was torn between feelings that she could have acted differently and yet when she returned to the moment and the factors that surrounded that particular birth also feeling that she could not have acted differently. Marie-Louise assists a woman who gives birth to her first baby. She finds it difficult to build a connection and create a relationship with the woman when her shift starts and the woman is already in the second stage. Afterwards she wishes she had asked the woman to change position to all fours which she thinks is preventive but explains that it would have been impossible.

*So I asked the obstetrician…do you think it would have made a difference if I had performed an episiotomy? I considered that but at the same time, I don’t believe in it being preventive. And she said ‘No, personally I don’t think so’, and I thought well, the perineum did actually had time to stretch, right… stupid perhaps that she was in lithotomy position. I wonder if that’s what did it. But trying to get her on all fours would have been out of the question. She never would have…she didn’t even have the strength to get down on the birth seat…*

### Coping with guilt and self-blame

The experience of guilt emerges from the midwives’ stories. It begins during the active phase of the second stage, before the injury, and continues afterwards in the encounter with the woman and the midwife’s colleagues and also when they subsequently reflect on the experience. How much guilt and self-blame the midwife feels varies. They relate both to the existing truth that sphincter injuries are avoidable but also to the fact that there are circumstances and factors that will affect whether the woman gets an injury.

*It’s always my responsibility to ensure they experience as little tearing as possible. That’s…that’s what I feel most responsibility for if it doesn’t work out. That’s the only thing I go home and beat myself up about. An emergency caesarean… well, there’s not much I can do about it… If I’m dealing with a shoulder dystocia, well, there’s actually nothing I can do to prevent that either…but the sphincter injuries are the only thing that I really, really think are my fault. Even if I…even though now that I am a relatively experienced midwife… I can actually feel that maybe I cannot always prevent this from happening…even if I do my very best…*

For some of the midwives, the feeling of guilt if woman gets an injury means they feel they have let the woman down. Vivian describes her encounter with a woman who is expecting her second child and had a sphincter injury when she gave birth the first time. Before meeting the woman, Vivian makes up a plan in her head for how to support the woman to give birth without sustaining a sphincter injury this time.

*Because I thought: Oh, a multip – wonderful! We’ll just breathe this baby out and I’ll be so precise and so careful…because I really wanted to do as good a job as possible.*

During the second stage the baby’s heartbeat drops and Vivian asks a colleague to assist and support her. Due to the baby’s heartbeat Vivian feels she needs to intervene and ask the woman push actively, which she sees as a risk factor in this case. The baby is born and is fine but the woman sustains a sphincter injury. Afterwards Vivian reflects upon her feelings of failure and of guilt towards the woman.

*Yes, but I feel I have failed as a midwife if she gets a torn sphincter… I mean, I feel ashamed! It’s appalling to call it ‘shame’, but that’s what it is. And I can’t be like some people, just shrug it off, saying ‘Yeah, yeah, well this happens sometimes’. No, I’m like ‘I have to make this work, dammit!’ You’re immediately thrown into a pit of uselessness… You are no good. No, but it feels like that. It’s sensitive…*

The midwives explain that the feelings of guilt, failure and disappointment differ depending on the circumstances that surround the birth. Ellen attends a birth where the woman is expecting her third child. Her first child was stillborn and this time the birth ends with a shoulder dystocia. A colleague of Ellen’s finally manages to resolve the shoulders and the baby is born and recovers after resuscitation. Both the parents, especially the father, panic during the last minutes of the birth, remembering and reliving the experience of their first baby’s birth. For the midwife Ellen the sphincter injury is not the primary focus in this case and she does not feel immense feelings of guilt.

*Yes…in this situation it felt like… it’s never fun and we’re always, always struggling to have as little tearing as possible, obviously. But in this case it…it somehow was allowed to be okay here anyway. We were all relieved the baby revived… In this case, the sphincter injury wasn’t the worst thing. No…the feelings in this case were connected to something much deeper…*

Since the midwife believes there is a consensus that a skilled midwife can prevent a sphincter injury from happening, she judges herself but is also afraid of being considered incompetent. Being a ‘bad’ midwife in this context would mean, in addition to not being able to prevent the injury, not having acted according to what is considered the right way to manage the second stage. The midwives are aware that the midwife who is responsible for the birth blames herself and a colleague is seldom or never criticized openly. It is perceived as a delicate matter to support a colleague while at the same time giving her feedback and discussing with her how she handled the situation.

*Sometimes, it is really our fault – if we force too much, raise the oxytocin drip too much, or too fast or something… anything like that. And then it actually is our fault, but we might never say so to a colleague… You don’t want to rub salt into the wound…I presume…Or maybe you don’t know the person well enough to criticise her. But then, it doesn’t really matter, 'cause everyone knows that it is indeed our fault sometimes…I think it’s more to show solidarity and try to soften the blow…*

Judging oneself is described by newly qualified midwives and by experienced ones. The less experienced midwives felt that experienced midwives do not blame themselves as much, wondering what would have happened if a more experienced colleague had attended the woman. A more experienced midwife on the other hand, has to deal with the fact that her experience was not enough to prevent the injury from happening.

*Everything changes, I believe, as you get more experience as a midwife… Because in a way sometimes I feel more today, that if the woman gets a sphincter injury I know that I’ve done everything I could… But at the same time I get more anxious because I think this shouldn’t have to happen to me anymore… Because surely I have enough experience now so that this should not happen! When I was newly qualified I attended a woman who got a sphincter injury and I know I thought it was simply awful, but when you’re green you are sort of forgiven. But as an experienced midwife I don’t see it like that…*

Feelings of guilt, shame and worrying that they may not having done everything possible to prevent the injury kept some of the midwives from sharing their feelings and reflecting upon the situation with other colleagues. They only revealed their true feelings and worries with someone they really trusted.

*But then…it’s not like I wouldn’t tell my colleagues what happened. I told them: 'OK, it would have been a fantastic birth if it wasn’t for this… she got a sphincter injury… and I’m not going to keep it quiet… but that I feel really bad about it, and the fact that I feel so ashamed – I’ll only tell Anna… But she is actually the only one…*

Even if the midwives felt hesitant to open up and share their feelings of failure and worries about not being competent they also acknowledged that there was support from colleagues if they wanted it. The support given was mostly compassion and sympathy and they were told that this happens to all midwives at times and that the midwife should reflect upon the situation and what she could have done differently but try not to blame herself. One of the midwives said that this is not the kind of support that she needs. Her experience is that there is no real reflection involved in the support given and she chose to not discuss the event with colleagues.

*When two midwives have gone through such a difficult situation together they sometimes support each other by saying ‘There, there, we did everything we could’. It could also just be a superficial thing, a way to avoid looking more deeply into oneself and questioning oneself. ‘Could we have done things differently?’ But it’s only… I’m only interested in that level of sincerity.*

### To accept, move on and forgive oneself

To be able to move on, the midwives tried to adopt different strategies such as reviewing the birth and the course of events, considering possible reasons for the injury and what they could have done differently and sometimes coming to the conclusion that they did the best they could under the circumstances.

*The feeling is different then because you feel that…you maybe, did what I believe…was the best thing and what I was able to do. I don’t really think…that any other midwife could have done things any better, I really don’t think so.*

In cases where the midwives felt very guilty they finally tried to accept that the fact that the woman suffered a sphincter injury and move on.

*Yeah, that’s the way our profession is. It’s unbelievably upsetting when you don’t, like, make it… but I can’t allow myself to bear the total responsibility… It’s completely…I shouldn’t ask myself to be able to cope with all situations and make all situations turn out happily… And make everybody happy and live for a thousand years… 'Cause that would somehow be like playing God. No, as a midwife you have to let go of that desire somehow. I won’t be able to connect equally well with everybody and I won’t do as good a job in all places… and I need to somehow be able to forgive myself for that…*

The midwives had to accept the frustration of having to move on without any definite answers as to why the injury happened or what they could have done to prevent it from happening, not even after reflecting upon the situation afterwards. Not having found a valid explanation for the injury also created a sense of being without strategies for the future if the situation were to happen again.

*Where…where sphincter injuries are concerned, I try to learn from the experience and still, or sometimes, like now, like this time, I feel that I don’t know what I possibly could have done differently.*

## Discussion

The main finding in this study is that the midwives relate to both an accepted truth that a skilled midwife can prevent an anal sphincter tear and at the same time to a more complex belief in relation to the phenomenon. Since the scientific evidence for the accepted truth is limited [9–11] the question is where this truth derives from. The problem of the increased incidence of OASIS was highlighted in the few first years after 2000. Among the affected professions there is an on-going discussion regarding the possible reasons for and the prevention of severe perineal trauma. Some obstetricians and midwives in Sweden argue that the increase in sphincter injuries is associated with midwives neglecting the importance of perineal protection and instead using hands-off techniques and the upright birth positions promoted by the natural birth movement of the 1990s [12, 13]. This view is supported mainly by observational studies [12, 13] but they may have had an impact on the accepted truth described by the midwives and contributed to their focus on the hands-on techniques. It is also possible that the accepted truth of midwives’ ability to avoid severe perineal trauma using hands-on techniques has been reinforced by discussions in the media.

If the scientific evidence is not consistent, perhaps the reason for this accepted truth that a skilled midwife can prevent sphincter injuries from happening, the focus on hands-on techniques and the importance of the hands-on techniques as a clinical skill could be explained by the concept of authoritative knowledge within obstetrics and midwifery culture? Authoritative knowledge is created within a community, in which the participants agree on what knowledge counts and is considered legitimate, consequential and official [14]. This is in line with the findings of this study as the accepted truth is regarded as more of a truth than is the more complex belief. However there seemed to be an uncertainty as to the consensus on the accepted truth whereby the midwives sometimes leaned more toward the more complex belief, which implies that sphincter injuries are not always possible to avoid. Altogether, this created a deadlock for the midwives that was difficult to resolve, which was expressed as an almost bodily feeling of being trapped when they recalled their experiences. In this situation of wanting to find answers and finding no definite answers, colleagues and cultural context are important. While collegial relationships can provide support and affirmation they are also a potential source of conflict [15]. These relationships and the midwife’s professional identity are so important that they are among the reasons why midwives stay in the profession [16].

The culture of midwifery has been described by Kirkham as a hierarchical culture, with a risk of vulnerability between midwives and pressure to conform [17]. This study was carried out in a context in which the midwife is in a continuous relationship with her colleagues rather than with the birthing woman, whom she only meets once. This could be the reason why the midwives expressed a sense not only of having failed the woman, but also of having failed professionally. The risk of being considered as not being competent may explain why midwives were hesitant to share their feelings of failure. This hesitancy may also have been intensified in this context as the midwives were evaluated in retrospect by their supervisors, which was perceived as an inadequate way to address the problem. There is a danger in a culture in which midwives hesitate to share feelings of guilt, self-blame and self-suspicion of not being competent enough. Not only will it create a sense of loneliness for the individual midwife but midwives may seek to appear competent because they lack the trust and confidence to discuss their lack of competence with colleagues [18].

The with-institution approach described by Hunter [15] may also explain the emphasis placed by the midwives in our study on the importance of managing the birth according to the best practice available and feeling less guilt if there was an acceptable reason why a sphincter injury occurred. Among acceptable reasons for a sphincter injury would include their having feared for the baby’s wellbeing, whereas a non-acceptable reason would be if a woman sustained an OASIS on the birth seat. In this setting, the midwife might blame herself if a woman incurred a sphincter injury on the birth seat, compared to if a woman incurred an injury in the lithotomy position, even though there is no scientific evidence to support this view [19, 20].

The findings of this study clearly show that the experience generated feelings of guilt and even shame among the midwives. Guilt, shame and pride are considered self-conscious emotions [21] and as such they contribute to promoting the stability of social hierarchies and affirm status roles [22]. Guilt and shame are feelings that are associated with experiences of not being sufficient and as moral feelings they are among our most private and most intimate ones [21]. Intimate feelings that are perceived as negative may not be easy to share with colleagues especially for those who struggle with the traditional image of perfection and striving for error-free practice that still lingers in both medicine and caring. Feelings of guilt and shame are described in medicine in relation to adverse outcome and error management [23] as for example, in a Swedish study on ambulance personnel’s experience of adverse outcomes. The authors concluded that most shameful thing was not failing in the care given to the patients but that the image the ambulance personnel had created of themselves, and for others to see, cracked [24]. This is in line with the findings of our study whereby guilt and shame were not only related to the woman but also to her perceived professional self-image.

Surprisingly, there were some issues potentially related to severe perineal trauma that were not mentioned by the midwives during the interviews. The woman and her ability to prevent a tear were topics largely absent from the discussions. Neither was the possibility of preventing the tear in collaboration with the woman mentioned often. The absence of the woman, where she is seen as passive and the caregiver as in control can be related to the with-institution ideology but is also described in Bergstrom’s study [25] of vaginal examinations during the second stage of labor, where multiple examinations were performed to teach the woman how to push correctly. The communication or lack thereof between the midwife and the mother was not discussed by the midwives in this study as much as perineal protection and birthing position. This was unexpected since our own experience is that midwives often talk about the importance of communication with the birthing woman. A protective factor in relation to perineal trauma is cooperation of the woman according to one study [26]. On the other, hand since the midwives were not specifically asked about their relationship and communication with the woman, this could explain why the midwives did not focus on that aspect of the birth. Since most of the midwives were prone to relate to the existing truth, their focus may primarily have been on what they themselves did or did not do as well as on their own feelings in relation to the experience rather than on relating to their relationship and collaboration with the woman.

The finding of this study implies the importance of further research on how to implement and evaluate reflective practices in midwifery care with the aim of professional development. Midwives describe sharing experiences as a way to develop professionally [27] and midwives need tools to enable them to discuss their experience openly and a working environment that encourages them to share feelings of uncertainty, distress and possible weaknesses as well as strengths, not only in relation to obstetric anal sphincter injuries but to all the difficulties a midwife can encounter.

An idea for future research is to study whether a concept of peer support during the second stage of labour could be implemented in a hospital-based setting. It would give midwives the opportunity to reflect on the management of the second stage together with a colleague, with the aim of professional development and with the potential of decreasing severe perineal trauma for the birthing women.

The strength of phenomenological research is the possibility to deepen the understanding of complex phenomena with all its variations and nuances. For this study a phenomenological approach was suitable due to the complexity of the studied phenomenon. However, phenomenological research results are always contextual and thus never to be understood as universal. Findings are applicable first of all in the same context [6]. On the other hand this does not entirely mean that the application of qualitative studies cannot be transformed to other contexts but if this is done the findings must be related to the new context [28]. In this study this means that the result must be related to being performed in Sweden with midwives working in urban areas in a high-technical hospital-based setting where they are expected to be able to care for two women in labour at the same time.

## Conclusions

This study highlights two coexisting beliefs among the midwives; the accepted truth in this context and a more complex beliefs; which together create a deadlock for the midwife difficult to resolve. Neither of the two beliefs is entirely supported by scientific evidence, which leaves the door open for a cultural explanation of why the truth is more regarded as a truth than is the more complex belief. This study indicates the importance of creating opportunities for reflection among midwives, and the necessity of creating a working culture that involves sharing experiences and includes examining feelings of fear, professional doubt and uncertainty.
